# Complete genome sequences of two *Gelderlandvirus* and one *Chivirus* bacteriophage from Burkina Faso wastewater, lytic against a foodborne *Salmonella enterica* subsp. *diarizonae* isolate

**DOI:** 10.1128/mra.00043-26

**Published:** 2026-03-10

**Authors:** Nafissatou Ouédraogo, Oumarou Soro, Vanessa Natasha, Moses Gachoya, Adama Fa Sere, Martin Georges, Nicolas Barro, Lillian Musila

**Affiliations:** 1 Département de Biochimie-Microbiologie, Université Daniel Ouezzin-Coulibaly, Dédougou, Burkina Faso; 2 Institute of Health Sciences, Erciyes University 52958, Kayseri, Turkey; 3 Microbiology Hub Kericho, Kenya Medical Research Institute/Walter Reed Army Institute of Research—Africa 118982, Nairobi, Kenya; 4 Laboratoire de Biologie Moléculaire, d’Épidémiologie et de Surveillance des Bactéries et Virus Transmissibles par les Aliments (LaBESTA), Université Joseph Ki-Zerbo 107750, Ouagadougou, Burkina Faso; Portland State University, Portland, Oregon, USA

**Keywords:** bacteriophages, *Gelderlandvirus*, *Chivirus*, *Salmonella enterica* subsp.* diarizonae*, Burkina Faso, genome sequence, wastewater

## Abstract

We report the genome sequences of three double-stranded DNA phages, from the *Gelderlandvirus* and *Chivirus* genera, isolated from wastewater in Burkina Faso, infecting a foodborne *Salmonella enterica* subsp. *diarizonae* isolate. Their genomes range from 59,952 to 161,888 bp, with a G+C content of 36.93–56.6% and 86–273 predicted coding sequences.

## ANNOUNCEMENT

*Salmonella enterica* subsp. *diarizonae* infects ectothermic animals but can cause human gastroenteritis and invasive infections (1, 2) via contaminated food and water (3). Bacteriophages (phages) are re-emerging as therapeutic and biocontrol agents (4).

Three phages, Femii, Zounloho, and Tiidero, were isolated from hospital wastewater (12°27′39.6″ N, 3°27′54.34812″ W) collected on 1 August 2024 in Dedougou, Burkina Faso, using a foodborne *S. enterica* subsp. *diarizonae* isolate as the host, through an enrichment method described elsewhere (5), with slight modifications. Briefly, 50 mL of wastewater was centrifuged (6,000 × *g*, 10 min) and filtered (0.45 μm). Ten milliliters of the supernatant was mixed with 10 mL of 1× tryptic soy broth (TSB; Oxoid Ltd., Basingstoke, England) and 100 μL of bacterial culture grown in 1× TSB. The mixture was incubated (37°C, 200 rpm, 24 h) and clarified through centrifugation. Phages were isolated using the double-layered (0.7% top/1.5% bottom) tryptic soy agar method and purified through three rounds of single plaque isolation (4). High-titer stocks were prepared for DNA extraction by infecting host cultures at a multiplicity of infection of 0.01 until complete lysis (4–6 h), followed by centrifugation and filtration (6). Host RNA and DNA were removed using RNase A and DNase I (Thermo Fisher Scientific, USA) (6). Phage DNA was extracted using the QIAamp Mini Kit (Qiagen, Germantown, MD, USA) following the manufacturer’s instructions.

Paired-end libraries (2 × 150 bp) were prepared using the Illumina DNA Prep (M) Tagmentation Kit (Illumina, San Diego, CA, USA) using the P1 (300) cycle reagent cartridge and sequenced on the Illumina NextSeq 1000. Raw reads were quality-checked with FastQC v0.12.1 (7), trimmed with fastp v0.23.4 (8), and assembled with SPAdes v4.2.0 (9) with --metaviral option. Bandage 0.8.1 (10) was used for assembly visualization, and sequence coverage was determined with BBMap v.39.27 (https://github.com/bbushnell/BBTools). PhageTerm v1.0.12 (11) predicted termini and CheckV v1.0.3 (12) confirmed 100% genome completeness. Genome annotation was performed using Pharokka 1.5.1 (13). Taxonomic classification based on Mash distance to the top hits was determined against the INPHARED database v1.8 (14). Complete phage genomes were screened using CRISPR-CasFinder v1.1.0 (15), CARD RGI v1.3.1 (16), and Virulence Factors Database (17) to determine CRISPR-like, AMR, and virulence genes, respectively. Bacphlip v.0.9.6 (18), PhaTYP (19), and PhageGE (20) consistently predicted a virulent lifestyle. tRNAs were predicted using tRNAscan-SE v2.0.12 (21). Sequence similarity searches were performed on BLAST (https://blast.ncbi.nlm.nih.gov/Blast.cgi). Default parameters were used for all software, except where otherwise noted.

**Fig 1:**
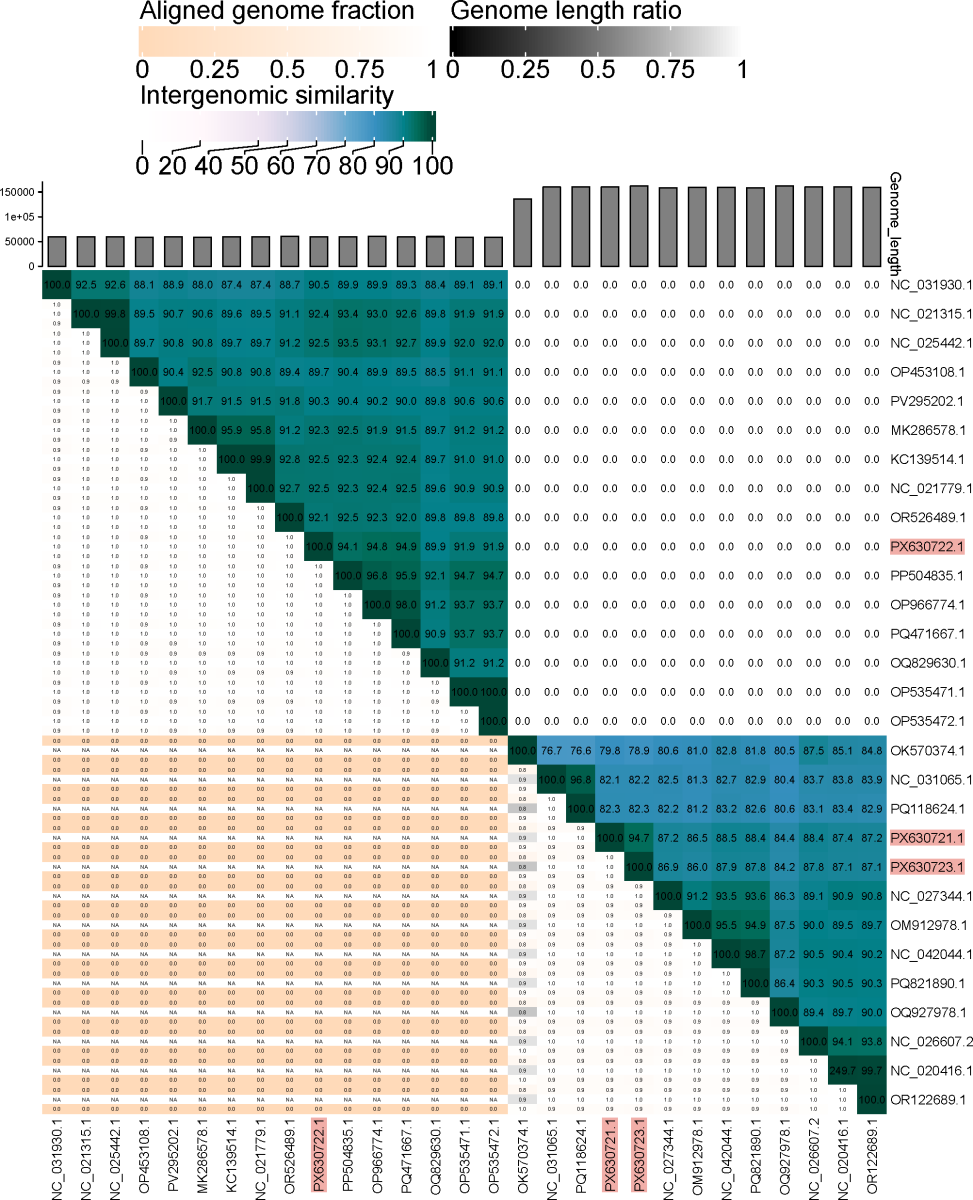
Heatmap generated by the Virus Intergenomic Distance Calculator (VIRIDIC) (22) showing pairwise intergenomic similarities, aligned genome fractions, and genome lengths for a subset of the most similar phages with a nucleotide sequence similarity of 95–99% to *Salmonella enterica* subsp. *diarizonae* phages Femii, Zounloho, and Tiidero. In the right half of the heatmap, the numbers represent the similarity values for each genome pair. The nucleotide identity of the complete genome length cutoff for genera (>70%) and species (>95%) (23) was used; phages with a similarity value of 0 belonged to different genera and species. In the left half, three indicator values are presented for each genome pair in the order from top to bottom: aligned fraction genome 1 (for the genome found in this row), genome length ratio (for the two genomes in this pair), and aligned fraction genome 2 (for the genome found in this column) (22). The GenBank accession numbers PX630721.1, PX630722.1, and PX630723.1 highlighted in red are those of the studied phages Femii, Zounloho, and Tiidero, respectively.

The NCBI database contains 11 *Gelderlandvirus* genomes similar to Femii, 9 to Tiidero, and 15 *Chivirus* genomes similar to Zounloho, all sharing 95–99% nucleotide sequence identity. Fig. 1 presents a VIRIDIC (22) heatmap of intergenomic similarities between these phages and the studied phages. Phage genomes ranged from 59,952 to 161,888 bp, with 36.93–56.58% G+C content and 86–273 coding sequences (Table 1). They are linear double-stranded DNA phages with 0–3 tRNA genes and no CRISPR-associated genes. All lacked antibiotic resistance, virulence, and lysogeny genes, indicating a strictly lytic lifecycle suitable for biocontrol of *S. enterica* subsp. *diarizonae*.

**TABLE 1:** Genomic characteristics of three *Salmonella enterica* subsp. *diarizonae* phages^*a*^

Phage name	BLASTn results		Family	Genus	Genome length (bp)	G+C content (%)	No. of CDS	CDS coding density	Genome coverage (X)	No. of raw reads	No. of tRNA	Termini	GenBank accession no.	SRA accession no.
	% identity top hit	Phage name (GenBank accession no.)												
Femii	97.92%	Phage STP4-a (NC_026607.2)	*Straboviridae*	*Gelderlandvirus*	160,006	36.93	271	96.01	4,158.59	2,325,603	3	Unknown	PX630721	SRR36255177
	97.62%	Phage vB_SenM-S16 (NC_020416.1)												
	97.62%	Phage vB_SenM-AKM_NP4 (OR122689.1)												
	97.58%	Phage Melville (NC_042044.1)												
Zounloho	97.22%	Phage vB_Sty_BCPSty_002 (PV295202.1)	*Casjensviridae*	*Chivirus*	59,952	56.58	86	97.64	27.90	1,929,293	0	Unknown	PX630722	SRR36255176
	97.09%	Phage HMD-P1 (PQ471667.1)												
	96.99%	Phage ST-W139 (OQ436961.1)												
	96.89%	Phage PST-D32 (OP966774.1)												
Tiidero	98.53%	Phage STP4-a (NC_026607.2)	*Straboviridae*	*Gelderlandvirus*	161,888	36.99	273	96.03	4,101.72	2,328,513	3	Cos (5')	PX630723	SRR36255175
	97.94%	Phage vB_SenM-S16 (NC_020416.1)												
	97.94%	Phage vB_SenM-AKM_NP4 (OR122689.1)												
	97.10%	Phage Melville (NC_042044.1)												

^*a*^ PhageTerm was unable to infer the genome termini for phages Femii and Zounloho likely due to the absence of a detectable read start bias or to circular permutation of the genomes. When tagmentation-based library preparation, such as Illumina DNA Prep (M) Tagmentation, is used, the physical ends of linear dsDNA molecules cannot be recovered, preventing PhageTerm from identifying the termini when terminase initiates DNA packaging at fixed positions (cos or pac) (11). Cos (5′), 5′ cohesive sequence; CDSs, coding sequences; SRA, Sequence Read Archive.

## Data Availability

The complete genome sequences of *Salmonella enterica* subsp. *diarizonae* phages are available in GenBank and in Sequence Read Archive under the accession numbers listed in Table 1. The *Salmonella enterica* subsp. *diarizonae* host strain used in this study is available from the corresponding author upon reasonable request.
